# High mortality and mechanical ventilation in COVID-19-associated pulmonary aspergillosis: insights from a two-center retrospective cohort study

**DOI:** 10.3389/fpubh.2025.1702502

**Published:** 2026-01-07

**Authors:** Aifang Zhong, Weijun Jiang, Yang Yang, Jun Hong, Qiuyue Wu, Lei Xiong, Li Han, Changjun Wang, Jiang Wu, Xinyi Xia

**Affiliations:** 1 Institute of Laboratory Medicine, Jinling Hospital, Affiliated Hospital of Medical School, Nanjing University, Nanjing, Jiangsu, China; 2 Medical Technical Support Division, The 904th Hospital, Changzhou, Jiangsu, China; 3 Department of Thoracic Surgery, Jinling Hospital, Nanjing University School of Medicine, Nanjing, China; 4 Center for Disease Control and Prevention of PLA, Beijing, China; 5 Department of Nuclear Medicine, Jinling Hospital, Affiliated Hospital of Medical School, Nanjing University, Nanjing, Jiangsu, China; 6 State Key Laboratory of Analytical Chemistry for Life Science, Nanjing University, Nanjing, China; 7 Jinling Hospital, The First School of Clinical Medicine, Southern Medical University, Nanjing, Jiangsu, China

**Keywords:** COVID-19 associated pulmonary aspergillosis, SARS-CoV-2, mortality, aspergillus, ICU

## Abstract

**Background:**

Studies on COVID-19-associated pulmonary aspergillosis (CAPA) have raised concerns regarding its high mortality rates. This study aims to assess the mortality of CAPA caused by Alpha and Omicron variants among intensive care unit (ICU) patients in China.

**Methods:**

We enrolled 236 ICU patients admitted to Wuhan Huoshenshan Hospital from 4 January 2020, to 30 March 2022, during the Alpha variant epidemic, and 187 ICU patients admitted to Nanjing Jinling Hospital from 24 October 2022, to 19 January 2023, during the Omicron variant epidemic. We systematically collected clinical data for analysis, and all patients were confirmed to have SARS-CoV-2 infection through polymerase chain reaction (PCR) testing.

**Results:**

The incidence of CAPA was 5.1% (12/236) during the Alpha variant period and increased to 7.5% (14/187) during the Omicron variant period. The mortality rate among CAPA cases was 33.3% (4/12) for patients infected with the Alpha variant and 28.6% (4/14) for those infected with the Omicron variant. Both rates were higher than the mortality rates among patients without CAPA, which were 4.9% (11/224) during the Alpha period and 8.7% (15/173) during the Omicron period. Mechanical ventilation in CAPA patients requires careful consideration. *Aspergillus fumigatus* was the most commonly identified causative agent in CAPA cases, while *Aspergillus flavus* was found in only one case.

**Conclusion:**

Our findings emphasize the necessity of implementing more effective mycological detection methods and promoting internationally recognized diagnostic criteria for CAPA.

## Introduction

Patients with viral pneumonia are at an increased risk of developing secondary bacterial and fungal infections, including invasive pulmonary aspergillosis (IPA), which is one of the most common life-threatening fungal infections in intensive care units (ICUs). Since the beginning of the pandemic, COVID-19-associated pulmonary aspergillosis (CAPA) has been increasingly reported, particularly in critically ill patients, raising concerns about its associated mortality rates. International experts have proposed consensus criteria for research and clinical guidance in defining and managing COVID-19-associated pulmonary aspergillosis (1). Nevertheless, the incidence and mortality rates of CAPA still exhibit significant variations due to differences in study design, medical settings, diagnostic methods, diagnostic criteria, and guidelines. Geographical variations in the incidence of CAPA have been noted, ranging from 3.3 to 38.7% (2). The review that encompassed 38 studies and 1,437 CAPA patients in the ICU found a median mortality rate of 56.8% (2, 3), with rates ranging from 30% in Portugal (4) to 91.8% in Pakistan (5). Although the true incidence remains uncertain, patients with CAPA have a significantly higher mortality rate than those without CAPA. A meta-analysis of 20 studies reported a mortality rate of 51.2% among CAPA patients, with an odds ratio for mortality of 2.83 when compared to controls (6). In a prospective cohort study involving 108 critically ill patients, the mortality rate was reported to be 44% for patients with CAPA compared to 19% for those without aspergillosis (7).

CAPA is often overlooked during diagnosis, resulting in a high rate of missed diagnoses. Autopsy studies have shown that IPA is one of the most common missed diagnoses in ICU admissions (8). Over the past 25 years, our institution has conducted 893 postmortem examinations of ICU patients. Out of these, 25 patients (2.8%) were diagnosed with invasive aspergillosis (IA) during the autopsy. Only 10 of these cases (40%) were classified as IA antemortem, based on the initiation of antifungal treatment (9). A systematic review of autopsy series involving individuals who died from COVID-19 revealed that 11 (2%) of 677 decedents had autopsy-proven invasive mold disease (IMD). This included eight cases of chronic pulmonary aspergillosis, two cases of unspecified IMD, and one case of disseminated mucormycosis (9). However, many patients with chronic pulmonary aspergillosis could not be classified according to the European Organisation for Research and Treatment of Cancer and Mycosis Study Group Education and Research Consortium (EORTC/MSG) criteria for invasive fungal disease (IFD). This difficult arises from a strict interpretation of host factors, which are suitable for patients who are immunosuppressed, particularly those with hematological malignancies or prolonged neutropenia.

On the other hand, the majority of CAPA patients also lack these classical host factors. This absence complicates the classification of CAPA patients using the same EORTC/MSG criteria. The 2020 European Confederation of Medical Mycology/International Society for Human and Animal Mycology (ECMM/ISHAM) consensus criteria incorporate guidelines for diagnosing SARS-CoV-2 infections and can effectively reduce the rate of missed diagnoses. Additionally, the performance of routine microbiological tests has been diminished in some medical centers due to the risk of nosocomial virus transmission among healthcare workers, resulting in limited microbial evidence available for diagnosing CAPA (10, 11).

The initial phase of the COVID-19 pandemic in 2020 was characterized by the prevalence of the Alpha variant. In contrast, the major epidemic outbreak in China in early 2022 was marked by the emergence and predominance of the Omicron variant. Therefore, we aimed to assess the incidence of CAPA in ICU patients infected with these strains and analyze the diagnostic approaches and management strategies used during their hospitalization.

## Materials and methods

### Study setting

This retrospective study was conducted at two tertiary hospitals in China: Wuhan Huoshenshan Hospital and Nanjing Jinling Hospital. Wuhan Huoshenshan Hospital was a specialized, temporary facility that was established at the onset of the pandemic in early 2020, dedicated exclusively to treating patients with COVID-19. Nanjing Jinling Hospital is a large, comprehensive teaching hospital with a long-standing ICU that manages COVID-19 patients alongside other critically ill patients during the subsequent Omicron wave. Including centers from different phases of the pandemic and with varying operational structures allows for a broader perspective of CAPA.

### Patients and clinical data

This retrospective study included 236 ICU patients with confirmed COVID-19, diagnosed through SARS-CoV-2 PCR, who were treated at Wuhan Huoshenshan Hospital from 4 January 2020 to 30 March 2022 during the Alpha variant epidemic. Additionally, it also included 187 ICU patients with confirmed COVID-19, diagnosed through SARS-CoV-2 PCR testing, who were treated at Nanjing Jinling Hospital from 24 October 2022, to 19 January 2023, during the Omicron variant epidemic.

We systematically extracted information from the electronic medical records, which included patient demographics (age and sex), underlying comorbidities (such as diabetes, heart diseases, pulmonary diseases, malignancies, and autoimmune diseases), details of clinical management (including the use and duration of corticosteroids, antibacterial and antifungal agents, and the occurrence of co-infections such as bacterial pathogens from the lower respiratory tract and bacteremia), laboratory results (specifically SARS-CoV-2 PCR), and comprehensive mycological data [including results from serum galactomannan, bronchoalveolar lavage (BAL) galactomannan, *Aspergillus* quantitative PCR (qPCR), and traditional culture].

### Study design

During the Alpha variant period, due to the urgency of the initial pandemic wave and limited medical resources, the only laboratory method available for detecting fungal infections was the serum galactomannan (GM) test. We performed serum GM testing on all ICU patients during the Alpha period, and cases that met the CAPA criteria were identified for further analysis. Invasive mechanical ventilation was defined as endotracheal intubation and mechanical ventilatory support for at least 24 h. This study was approved by the Medical Ethical Committee of Wuhan Huoshenshan Hospital (HSSLL011) and the Ethical Review Committee of Nanjing Jinling Hospital (2023JLHGZRDWLS-00042).

### Definitions for CAPA

CAPA was defined in accordance with the 2020 ECMM/ISHAM consensus criteria. The entry criterion required a positive SARS-CoV-2 RT-PCR test at any time between hospital admission and ICU admission or within 72–96 h following ICU admission. Proven CAPA necessitates histopathological or direct microscopic detection of fungal hyphae or a positive culture or PCR for *Aspergillus* obtained from a sterile specimen via pulmonary aspiration or biopsy. Probable CAPA was identified by radiological findings (such as pulmonary infiltrates or a cavitary lesion not attributed to another cause), along with at least one of the following criteria: microscopic detection of fungal elements in BAL, a positive *Aspergillus* culture from BAL, a serum GM index of >0.5, or a BAL GM index of ≥1.0. Possible CAPA was identified by compatible radiological features along with at least one of the following criteria: fungal elements indicating a mold in non-bronchoscopic lavage (NBL), a positive *Aspergillus* culture from NBL, a single NBL GM index of >4.5, or an NBL GM index of >1.2 on two or more occasions. The *Aspergillus* in Intensive Care Unit (AspICU) clinical algorithm was utilized as a comparative diagnostic criterion for pulmonary aspergillosis (12).

### Statistical analysis

Descriptive and comparative analyses were performed. Considering the small sample size and non-parametric distribution of certain variables, Fisher’s exact test was applied to categorical variables, and the Mann–Whitney *U* test was used for continuous variables. A *p*-value of <0.05 was deemed statistically significant.

## Results

### Classification of CAPA

Serum GM testing was conducted on all 236 ICU patients, and 12 of them tested positive. Samples from these 12 patients were then sent to an external testing institution for qPCR and traditional culture analysis. These 12 patients were diagnosed with CAPA and underwent a detailed analysis. In the Omicron variant period, the medical records of all 187 ICU patients were searched for *Aspergillus*-related terms, and 14 patients who met the criteria for CAPA were identified.

**Figure 1:**
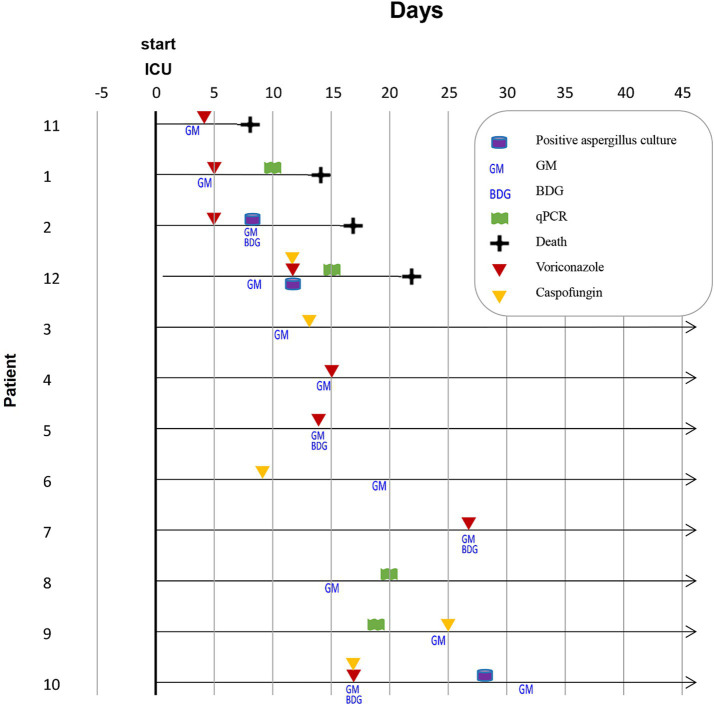
Timeline of mycological diagnostics in 12 CAPA cases caused by the Alpha variant. ICU: intensive care unit, GM: galactomannan, BDG: 1–3-*β*-D-glucan.

**Figure 2:**
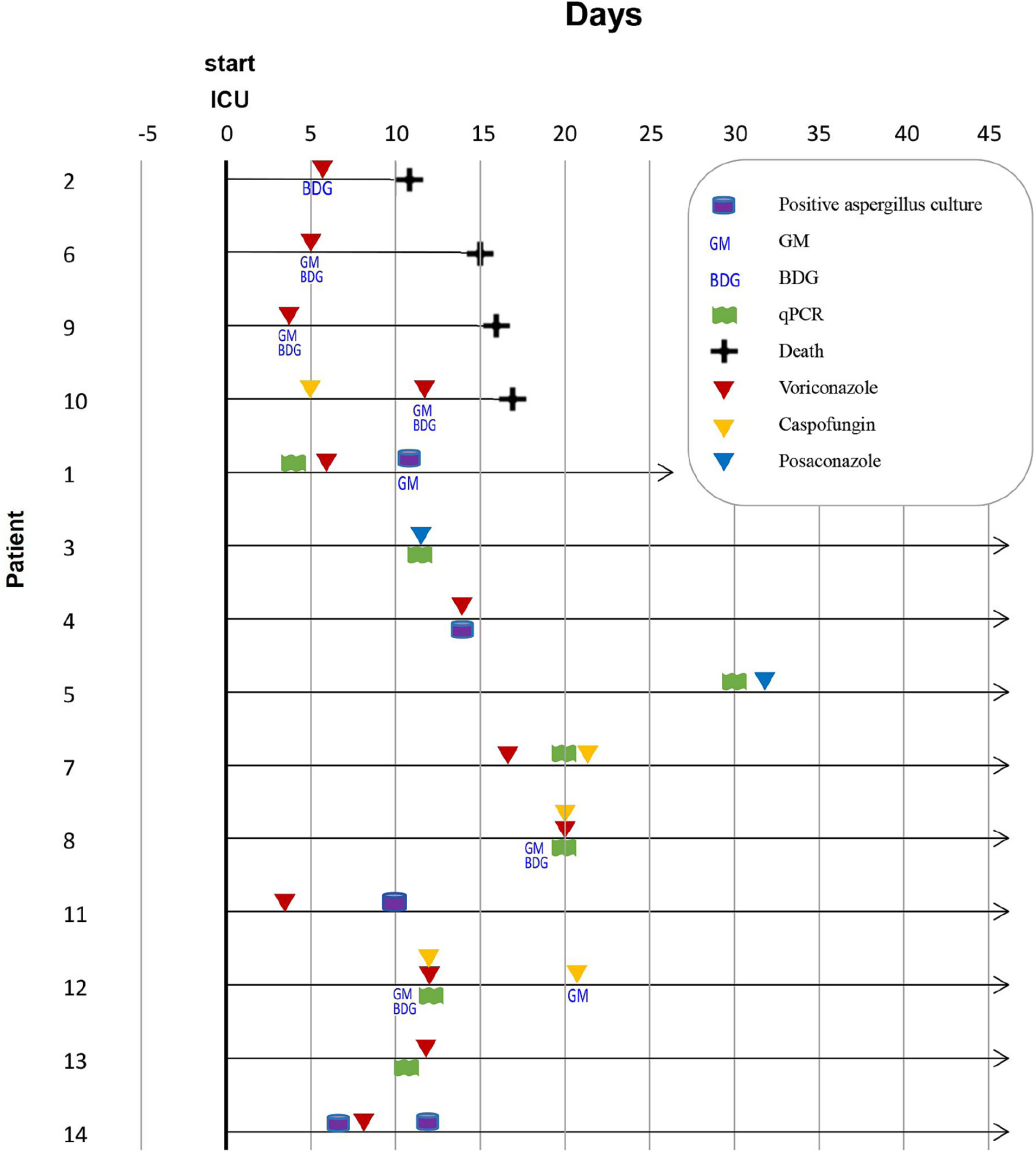
Timeline of mycological diagnostics in 14 CAPA cases caused by the Omicron variant. ICU: intensive care unit, GM: galactomannan, BDG: 1–3-β-D-glucan.

In our study, the incidence of CAPA was 5.1% (12/236) during the Alpha variant period (Figure 1; Table 1) and increased to 7.5% (14/187) during the Omicron variant period (Figure 2; Table 2). The mortality rate among CAPA cases was 33.3% (4/12) during the Alpha period and 28.6% (4/14) during the Omicron period (Table 3). Based on the 2020 ECMM/ISHAM consensus criteria, all 12 cases (100%) during the Alpha period were classified as probable CAPA, whereas 11 cases (78.6%) during the Omicron period were classified as probable CAPA, and 3 cases (21.4%) were classified as possible CAPA.

**Table 1:** Diagnostic criteria of CAPA caused by the Alpha variant.

	Mycological results														
Case number	Positive *Aspergillus* culture in BAL	Positive *Aspergillus* culture in NBL	Positive direct examination of BAL or NBL showing hyphae	BAL galacto-mannan	NBL galacto-mannan	Serum galacto-mannan	qPCR	AspICU algorithm(1, 2)					2020 ECMM/ISHAM criteria for CAPA(1)		
								Host factors	Risk factors	Clinical features	Imaging features	IPA definition	Host factors	Clinical features	CAPA definition
1						X	X		X	X	X	Probable	X	X	Probable
2		X				X			X	X	X	Probable	X	X	Probable
3						X			X	X	X	PAoAC	X	X	Probable
4						X			X	X	X	PAoAC	X	X	Probable
5						X		X	X	X	X	Probable	X	X	Probable
6						X			X	X	X	PAoAC	X	X	Probable
7						X			X	X	X	PAoAC	X	X	Probable
8						X	X		X	X	X	PAoAC	X	X	Probable
9						X	X	X	X	X	X	Probable	X	X	Probable
10						X			X	X	X	PAoAC	X	X	Probable
11						X			X	X	X	PAoAC	X	X	Probable
12		X				X	X		X	X	X	Probable	X	X	Probable

BAL, bronchoalveolar lavage; NBL, non-bronchoscopic lavage; ECMM, European Confederation for Medical Mycology; ISHAM, International Society for Human and Animal Mycology; CAPA, COVID-associated pulmonary aspergillosis; IPA, invasive pulmonary aspergillosis; PAoAC, possible aspergillosis or Aspergillus colonization.

**Table 2:** Diagnostic criteria of CAPA caused by the Omicron variant.

	Mycological results														
Case number	Positive *Aspergillus* culture in BAL	Positive *Aspergillus* culture in NBL	Positive direct examination of BAL or NBL showing hyphae	BAL galacto-mannan	NBL galacto-mannan	Serum galacto-mannan	qPCR	AspICU algorithm(12)					2020 ECMM/ISHAM criteria for CAPA(1)		
								Host factors	Risk factors	Clinical features	Imaging features	IPA definition	Host factors	Clinical features	CAPA definition
1		X		X		X	X	X	X	X	X	Probable	X	X	Probable
2							X		X	X	X	PAoAC	X	X	Probable
3							X		X	X	X	PAoAC	X	X	Probable
4		X							X	X	X	PAoAC	X	X	Possible
5							X		X	X	X	PAoAC	X	X	Probable
6						X			X	X	X	PAoAC	X	X	Probable
7							X	X	X	X	X	Probable	X	X	Probable
8						X	X		X	X	X	Probable	X	X	Probable
9						X		X	X	X	X	Probable	X	X	Probable
10						X			X	X	X	PAoAC	X	X	Probable
11		X						X	X	X	X	PAoAC	X	X	Possible
12						X	X		X	X	X	Probable	X	X	Probable
13							X		X	X	X	PAoAC	X	X	Probable
14		X							X	X	X	PAoAC	X	X	Possible

BAL, bronchoalveolar lavage, NBL, non-bronchoscopic lavage, ECMM, European Confederation for Medical Mycology, ISHAM, International Society for Human and Animal Mycology, CAPA, COVID-associated pulmonary aspergillosis, IPA, invasive pulmonary aspergillosis, PAoAC, possible aspergillosis or Aspergillus colonization.

**Table 3:** Factors associated with CAPA mortality.

Variable	Alpha variant				Omicron variant			
	Total (*n* = 12)	Survivor (*n* = 8)	Non-survivor (*n* = 4)	*p*-value	Total (*n* = 14)	Survivor (*n* = 10)	Non-survivor (*n* = 4)	*p*-value
General host factors								
Age median (range) years	67 (56–87)	63 (56–71)	77 (57–87)	0.139^U^	68 (37–81)	68 (37–81)	68 (47–81)	0.723^U^
Female sex	17%	25%	0	0.515^F^	36%	30%	50%	0.580^F^
Comorbidities								
Diabetes	25%	25%	25%	1.000^F^	36%	50%	0	0.221^F^
Heart diseases	17%	13%	25%	1.000^F^	43%	40%	50%	1.000^F^
Pulmonary diseases	17%	25%	0	0.515^F^	29%	30%	25%	1.000^F^
Malignancies	8%	13%	0	1.000^F^	21%	20%	25%	1.000^F^
Autoimmune diseases	0	0	0		21%	10%	50%	0.176^F^
Clinical condition and management prior to CAPA diagnosis								
Mechanical ventilation	33%	13%	75%	0.067^F^	43%	20%	100%	0.015^F^
Lower respiratory tract bacterial infection or colonization	17%	13%	25%	1.000^F^	43%	30%	75%	0.245^F^
Bacteremia	17%	0	50%	0.091^F^	21%	10%	50%	0.176^F^
Corticosteroids	100%	100%	100%		100%	100%	100%	
Antibiotics	75%	63%	100%	0.491^F^	93%	90%	100%	1.000^F^
Mycological diagnostics of CAPA cases								
*Aspergillus* species								
*Aspergillus fumigatus*	42%	38%	50%	1.000^F^	93%	90%	100%	1.000^F^
*Aspergillus flavus*	0	0	0		7%	10%	0	1.000^F^
Other or unknown	58%	63%	50%	1.000^F^	0	0	0	
Serum GM Index > 0.5	100%	100%	100%		43%	30%	75%	0.245^F^
BAL GM Index ≥ 1.0	0	0	0		7%	10%	0	1.000^F^
2020 ECMM/ISHAM CAPA classification								
Probable CAPA	100%	100%	100%		79%	80%	75%	1.000^F^
Possible CAPA	0	0	0		21%	20%	25%	1.000^F^
MB-AspICU IPA classification								
Probable IPA	42%	25%	75%	0.222^F^	36%	40%	25%	1.000^F^
Possible IPA or *Aspergillus* colonization	58%	75%	25%	0.222^F^	64%	60%	75%	1.000^F^
CAPA treatment								
Voriconazole	33%	38%	25%	1.000^F^	57%	40%	75%	0.559^F^
Caspofungin	25%	38%	0	0.490^F^	0	0	0	
Posaconazole	0	0	0		14%	20%	0	1.000^F^
Voriconazole + caspofungin	17%	13%	25%	1.000^F^	21%	30%	25%	1.000^F^

The cut-off values for GM were adopted from the ECMM/ISHAM CAPA classification. ICU, intensive care unit, GM, galactomannan, BAL, bronchoalveolar lavage, ECMM, European Confederation for Medical Mycology, ISHAM, International Society for Human and Animal Mycology, CAPA, COVID-associated pulmonary aspergillosis, U, Mann–Whitney U test, F, Fisher’s exact test.

Using the AspICU algorithm, five cases (41.7%) during the Alpha period were identified as probable IPA, with the remaining seven cases (58.3%) categorized as possible IPA or *Aspergillus* colonization. During the Omicron period, five cases (35.7%) were identified as probable IPA, and the remaining nine cases (64.3%) were categorized as possible IPA or *Aspergillus* colonization. These findings indicated that employing various criteria results in significant differences in the classification of CAPA. Adhering to the 2020 ECMM/ISHAM consensus criteria may improve the accuracy of CAPA diagnosis, thereby facilitating timely antifungal treatment.

### Detailed description of CAPA cases caused by the Alpha variant

Serum GM tests were performed on all 236 ICU patients, and 12 cases were found to be positive. Samples from these 12 patients were sent for qPCR and traditional culture analysis. No BAL or NBL samples were obtained. The majority of patients were men (*n* = 10, 83.3%) with a median age of 67 years (range: 56–78 years). The most common comorbidities were diabetes (*n* = 3, 25.0%), followed by heart diseases (*n* = 2, 16.7%), pulmonary diseases (*n* = 2, 16.7%), and malignancy (*n* = 1, 8.3%). All patients (*n* = 12, 100%) received corticosteroid therapy during ICU hospitalization.

Bacterial pathogens from the lower respiratory tract were isolated in two patients, both of whom had *Klebsiella pneumoniae* infection. One of these patients experienced bacteremia. Additionally, two other patients were suspected of having *Candida albicans* infection (Table 3; Supplementary Table S1). Nine patients (75.0%) received antibacterial treatment. *Aspergillus fumigatus* was identified in five patients (41.7%), while the species was unknown in the remaining seven patients.

The most commonly used antifungal drugs included voriconazole (*n* = 4, 33.3%), caspofungin (*n* = 3, 25.0%), and voriconazole and caspofungin combination therapy in two patients (16.7%). Detailed clinical and radiological characteristics are presented in Supplementary Table S1. Three of four (75%) non-survivors with CAPA received mechanical ventilation, while 10 of 11 (91%) non-survivors without CAPA received mechanical ventilation (Supplementary Table S2).

### Detailed description of CAPA cases caused by the Omicron variant

Among the 14 CAPA cases identified during the Omicron period, qPCR (*n* = 8, 57.1%) and serum GM testing (*n* = 6, 42.9%) were the most commonly used mycological detection methods. The majority of patients were men (*n* = 9, 64.3%) with a median age of 68 years (range: 37–81 years). The most common comorbidities were heart diseases (*n* = 6, 42.9%), followed by diabetes (*n* = 5, 35.7%), pulmonary diseases (*n* = 4, 28.6%), malignancy (*n* = 3, 21.4%), and autoimmune disease (*n* = 3, 21.4%). Six patients (42.9%) received invasive mechanical ventilation, and all four non-survivors (100%) received invasive mechanical ventilation. Out of 15 patients, 10 (67%) non-survivors without CAPA received mechanical ventilation (Supplementary Table S3).

A significant difference in invasive mechanical ventilation use (*p* = 0.015) was observed between survivors and non-survivors. All patients (*n* = 14, 100%) received corticosteroid therapy during hospitalization. Bacterial pathogens from the lower respiratory tract were isolated from six patients: two infected with *Acinetobacter baumannii*, two with *Pseudomonas aeruginosa*, and two with *Pneumocystis jirovecii*. One other patient had *Candida albicans* infection. A total of 13 patients (92.9%) received antibacterial treatment. *Aspergillus fumigatus* was identified in 13 patients (92.9%), and only 1 case (7.1%) had *Aspergillus flavus* infection.

The most commonly used antifungal drugs were voriconazole (*n* = 8, 57.1%), posaconazole (*n* = 2, 14.3%), and voriconazole and caspofungin in three patients (*n* = 3, 21.4%). Detailed clinical characteristics are presented in Supplementary Table S4.

## Discussion

Given that SARS-CoV-2 continues to circulate, awareness of invasive fungal infections in some settings remains insufficient. Our study highlights the significant burden of CAPA in ICU patients with COVID-19 and underscores the need to establish internationally recognized diagnostic criteria to guide effective prevention and management strategies.

In this two-center retrospective ICU patient study, the incidence of CAPA during the Alpha and Omicron periods was 5.1 and 7.5%, respectively, which is within the range reported in other studies (0.9–38%) (13–17). Variation in incidence is likely attributable to the study design, patient heterogeneity, diagnostic methods, and diagnostic criteria. The mortality rates among CAPA patients were 33.3% (4/12) for the Alpha variant and 28.6% (4/14) for the Omicron variant. These rates were similar between the variants and significantly higher than those of patients without CAPA [4.9% (11/224) for the Alpha variant and 8.7% (15/173) for the Omicron variant]. A systematic review of 20 studies found a pooled mortality rate of 51.2%, ranging from 15% (18) to 75.5% (19, 20).

Despite growing concerns, the actual prevalence of CAPA among ICU patients may be underestimated, mainly because of inadequate screening and inconsistent diagnostic criteria. Inconsistent case definitions may lead to overlooked or misclassified CAPA cases, delayed timely antifungal therapy, and unnecessary treatment, thereby contributing to poor outcomes. One study reported a 15% prevalence of CAPA using the 2020 ECMM/ISHAM criteria, which decreased to 11% when reclassified using the AspICU criteria (21). Magdalena et al. reported a lack of proven CAPA cases and a low percentage of probable cases (25%) despite a high mortality rate (76.5%), suggesting a potential underdiagnosis of CAPA (22).

Furthermore, during the early stages of the Alpha period, mycological examinations were largely restricted to ICU patients due to limited medical resources. Consequently, many non-ICU patients did not undergo comprehensive mycological examinations, potentially resulting in underdiagnosis.

Moreover, the difficulty in obtaining and testing specific samples also contributes to a lower CAPA diagnosis rate. Obtaining biological specimens, especially BAL samples obtained through bronchoscopy, can pose risks to patients and staff. This procedure is considered an aerosol-generating activity and is often minimized during pandemics (17). Studies have shown that the sensitivity (90%) and specificity (90%) of GM testing in BAL exceed those of serum GM testing (sensitivity ~40–70%, specificity ~85–90%) and culture methods (sensitivity ~45–60%) (23, 24). Limited bronchoscopy availability and inadequate access to diagnostic biomarkers, such as BAL GM, may result in the underestimation of the incidence (25). In our study, no lung tissue or BAL samples were available for Alpha-variant CAPA cases. Only one BAL sample was obtained for GM testing among the Omicron variant CAPA cases.

Traditional diagnostic criteria for invasive fungal diseases were primarily developed for patients with compromised immune systems and may be less suitable for critically ill COVID-19 patients. Several factors contributed to this phenomenon. First, neutropenia, hematologic malignancies, and prolonged corticosteroid therapy are major host factors that predispose patients to invasive *Aspergillus* infections (26, 27). In contrast, the majority of COVID-19 patients lack these classical host factors. Second, bronchoscopy is often inaccessible to ensure a reliable sampling. Third, clinical presentations and imaging findings often overlap with those of SARS-CoV-2 infection, with many patients lacking distinctive radiological manifestations due to concurrent mechanical ventilation or COVID-19 pneumonia. The 2020 ECMM/ISHAM definitions include COVID-19 patients requiring intensive care as a host factor, necessitating a temporal association. These criteria broaden the scope of diagnosing CAPA and effectively reduce missed diagnoses. The classification of possible CAPA should be considered sufficient for initiating antifungal therapy.

Our data showed an association between mechanical ventilation and higher mortality in both CAPA and non-CAPA patients. Although establishing a causal relationship between mechanical ventilation and increased mortality, specifically in CAPA, is challenging, mechanical ventilation is a known risk factor for secondary infections, including CAPA. Therefore, its use in patients with CAPA warrants careful consideration alongside the prompt initiation of appropriate antifungal therapy. Given the high CAPA-related mortality, empirical antifungal treatment for *Aspergillus* prior to positive mycological results was initiated in some cases. While the selection of antifungal drugs is important, timely initiation is crucial for improving prognosis (28).

This study has several limitations. First, there were no proven CAPA cases, primarily because of the lack of lung tissue biopsies. Second, serum GM testing, a primary diagnostic tool, is typically performed only once, limiting its sensitivity; serial testing (e.g., twice weekly) is recommended for improved detection. Third, mycological detection methods are limited, particularly with regard to culture, which potentially leads to suboptimal classification. Fourth, as CAPA occurs predominantly in ICU patients, this study focused only on ICU patients, omitting non-ICU patients, which limits the generalizability of findings and restricts the exploration of CAPA in less severe cases. Finally, while the inclusion of two distinct centers from different epidemic waves enhances the diversity of our cohort, specific clinical settings and patient populations may affect the external generalizability of our findings. Larger prospective studies including non-ICU patients from diverse geographic regions are required.

## Conclusion

This study analyzed data from two distinct centers across different waves of variant predominance, allowing for a comparison of CAPA features during the periods of Alpha and Omicron dominance. It also assessed compliance with various diagnostic criteria. Given the high mortality rates, it is crucial to raise awareness among clinicians about CAPA, implement more effective and accessible mycological detection methods, such as serial GM testing and BAL, where possible, and encourage the use of internationally recognized diagnostic criteria, including the ECMM/ISHAM standards.

## Data Availability

The original contributions presented in the study are included in the article/Supplementary material, further inquiries can be directed to the corresponding authors.
