# Supplementary material for: High mortality and mechanical ventilation in COVID-19-associated pulmonary aspergillosis: insights from a two-center retrospective cohort study
Source: Front Public Health. 2026 Jan 7;13:1702502. doi: 10.3389/fpubh.2025.1702502 (PMC12819614; doi:10.3389/fpubh.2025.1702502)
Supplement: Supplementary file 1 [file Supplementary_file_1.docx]

| **Case Number** | **Sex/Age** | **Underlying**  **diseases** | **Clinical data** | **Radiological data** | **CAPA classification** | **Other microbial infections** | **Outcome** |
| --- | --- | --- | --- | --- | --- | --- | --- |
| 1 | M/73 | none | fever | bilateral patchy areas of ground-glass opacities | probable | *Klebsiella pneumoniae* | death |
| 2 | M/87 | diabetes,  chronic kidney disease | fever,  worse  ventilatory | bilateral patchy areas of ground-glass opacities，enveloping pleural effusion | probable | *Candida albicans* | death |
| 3 | M/63 | none | fever,  worse  ventilatory | bilateral patchy areas of pleural effusion | probable | *Klebsiella pneumoniae* | alive |
| 4 | M/56 | none | worse  ventilatory | bilateral patchy areas of ground-glass opacities | probable | none | alive |
| 5 | F/63 | lung cancer with brain metastasis | fever,  worse  ventilatory | [space-occupying lesion in left upper lobe](http://dict.cn/Space-occupying%20lesion%20in%20left%20upper%20lobe%20of%20lung) | probable | none | alive |
| 6 | M/62 | diabetes,  hypertension | fever,  worse  ventilatory | bilateral patchy areas of ground-glass opacities，pneumothorax | probable | none | alive |
| 7 | M/75 | diabetes,  hypertension,  coronary heart disease | fever,  worse  ventilatory | bilateral patchy areas of multiple patchy exudative lesions | probable | none | alive |
| 8 | M/56 | none | fever,  worse  ventilatory | bilateral fibrosis | probable | none | alive |
| 9 | F/70 | breast cancer,  hypertension,  type I respiratory failure | worse  ventilatory | extensive faint exudation, white lung | probable | *Candida albicans* | alive |
| 10 | M/71 | none | fever,  worse  ventilatory | bilateral patchy areas of ground-glass opacities | probable | none | alive |
| 11 | M/57 | none | worse  ventilatory | bilateral patchy areas of ground-glass opacities，pneumothorax | probable | none | death |
| 12 | M/81 | coronary heart disease,cerebral infarction, Alzheimer's disease | fever,  worse  ventilatory | bilateral patchy areas of ground-glass opacities | probable | none | death |

**Supplementary Table S1.** Characteristics of CAPA patients caused by alpha variant.

CAPA: COVID-19 associated pulmonary aspergillosis; M: male; F: female.

**Supplementary Table S2.** The association of mechanical ventilation with CAPA-related mortality and non-CAPA-related mortality of alpha variant.

| **Factor** | **Alpha strain (n=236)** | | | | | | |
| --- | --- | --- | --- | --- | --- | --- | --- |
|  | **CAPA (n=12)** | | | | **Non-CAPA (n=224)** | | |
|  | **Survivor**  **(n=8)** | **Non-suvivor**  **(n=4)** | **P-value** | **Survivor**  **(n=213)** | | **Non-suvivor**  **(n=11)** | **P-value** |
| Mechanical ventilation | 13% (1/8) | 75% (3/4) | 0.0667^F^ | 3% (7/213) | | 91% (10/11) | ＜0.0001^F^ |

CAPA: COVID-19 associated pulmonary aspergillosis; F—Fisher’s Exact Test.

**Supplementary Table S3.** The association of mechanical ventilation with CAPA-related mortality and non-CAPA-related mortality of omicron variant.

| **Factor** | **Omicron strain (n=187)** | | | | | |
| --- | --- | --- | --- | --- | --- | --- |
|  | **CAPA (n=14)** | | | **Non-CAPA (n=173)** | | |
|  | **Survivor**  **(n=10)** | **Non-suvivor**  **(n=4)** | **P-value** | **Survivor**  **(n=158)** | **Non-suvivor**  **(n=15)** | **P-value** |
| Mechanical ventilation | 20% (2/10) | 100% (4/4) | 0.0150^F^ | 10% (16/158) | 67% (10/15) | ＜0.0001^F^ |

CAPA: COVID-19 associated pulmonary aspergillosis; F—Fisher’s Exact Test.

| **Case Number** | **Sex/Age** | **Underlying**  **diseases** | **Clinical data** | **Radiological data** | **CAPA classification** | **Other microbial infections** | **Outcome** |
| --- | --- | --- | --- | --- | --- | --- | --- |
| 1 | M/69 | diabetes,  hypertension,  coronary heart disease | fever | bilateral patchy areas of ground-glass opacities，enveloping pleural effusion | Probable | *Candida albicans* | alive |
| 2 | M/73 | renal insufficiency,  chronic obstructive pulmonary disease | fever,  worse  ventilatory | bilateral patchy areas of ground-glass opacitie | Probable | *Acinetobacterbaumannii* | death |
| 3 | M/81 | diabetes,  hypertension, | fever | bilateral patchy areas of pleural effusion | Probable | none | alive |
| 4 | M/74 | bronchial asthma,  chronic obstructive pulmonary disease,  heart disease | fever | bilateral patchy areas of ground-glass opacitie, emphysema,  Bilateral several small nodules | Possible | none | alive |
| 5 | M/69 | diabetes,  hypertension,  kidney cancer,  urothelial carcinoma of bladder,  obesity | none | bilateral patchy areas of multiple patchy exudative lesions | Probable | *Acinetobacterbaumannii* | alive |
| 6 | M/81 | pulmonary fibrosis | fever,  worse  ventilatory | bilateral fibrosis,  micronodules | Probable | *Pseudomonas aeruginosa* | death |
| 7 | F/67 | diabetes,  hypertension,  chronic obstructive pulmonary disease,  lung cancer | worse  ventilatory | occupying shadow in left lobe | Probable | *Pseudomonas aeruginosa* | alive |
| 8 | F/47 | respiratory failure, renal insufficiency, hepatitis B infection | fever | bilateral patchy areas of ground-glass opacitie,  pleural effussion | Probable | *Pneumocystis yersoni* | alive |
| 9 | F/63 | hypertension,  small intestine tumor | fever | diffuse reticular  and alveolar  opacities | Probable | none | death |
| 10 | F/47 | ANCA-associated vasculitis | fever,  worse  ventilatory | consolidations,  bilateral patchy areas of ground-glass opacitie | Probable | *Pneumocystis yersoni* | death |
| 11 | M/37 | myelosuppression after chemotherapy,  invasive pulmonary aspergillosis | fever | Plenty of pleural fluid on the left, bilateral peribronchovascular consolidations, nodules | Possible | none | alive |
| 12 | M/56 | diabetes,  hypertension,  cerebral infarction | fever,  worse  ventilatory | bilateral patchy areas of ground-glass opacitie | Probable | none | alive |
| 13 | F/61 | none | fever,  worse  ventilatory | bilateral patchy areas of ground-glass opacitie, left lung necrotic cavity | Probable | none | alive |
| 14 | M/70 | chronic heart failure | fever,  worse  ventilatory | bilateral patchy areas of ground-glass opacitie | Possible | none | alive |

**Supplementary Table S4.** Characteristics of CAPA patients caused by omicron variant.

CAPA: COVID-19 associated pulmonary aspergillosis; M: male; F: female.
